# Validation of CSR model to predict stroke risk after transient ischemic attack

**DOI:** 10.1038/s41598-021-04405-2

**Published:** 2022-01-12

**Authors:** Lu Zhao, Shuang Cao, Lulu Pei, Hui Fang, Hao Liu, Jun Wu, Shilei Sun, Yuan Gao, Bo Song, Yuming Xu

**Affiliations:** 1 Department of Neurology, The First Affiliated Hospital of Zhengzhou University, Zhengzhou, Henan People’s Republic of China; 2 Department of Magnetic Resonance, The First Affiliated Hospital of Zhengzhou University, Zhengzhou, Henan People’s Republic of China

**Keywords:** Neurology, Risk factors

## Abstract

It is essential to identify high risk transient ischemic attack (TIA) patients. The previous study reported that the CSR (comprehensive stroke recurrence) model, a neuroimaging model, had a high predictive ability of recurrent stroke. The aims of this study were to validate the predictive value of CSR model in TIA patients and compare the predictive ability with ABCD^3^-I score. Data were analyzed from the prospective hospital-based database of patients with TIA which defined by the World Health Organization time-based criteria. The predictive outcome was stroke occurrence at 90 days. The receiver-operating characteristic (ROC) curves were plotted and the C statistics were calculated as a measure of predictive ability. Among 1186 eligible patients, the mean age was 57.28 ± 12.17 years, and 474 (40.0%) patients had positive diffusion-weighted imaging (DWI). There were 118 (9.9%) patients who had stroke within 90 days. In 1186 TIA patients, The C statistic of CSR model (0.754; 95% confidence interval [CI] 0.729–0.778) was similar with that of ABCD^3^-I score (0.717; 95% CI 0.691–0.743; Z = 1.400; *P* = 0.1616). In 474 TIA patients with positive DWI, C statistic of CSR model (0.725; 95% CI 0.683–0.765) was statistically higher than that of ABCD^3^-I score (0.626; 95% CI 0.581–0.670; Z = 2.294; *P* = 0.0245). The CSR model had good predictive value for assessing stroke risk after TIA, and it had a higher predictive value than ABCD^3^-I score for assessing stroke risk for TIA patients with positive DWI.

## Introduction

Transient ischemic attack (TIA) is one of the most common acute cerebrovascular disease and is associated with high risk of early stroke^1,2^. Therefore, it is essential to identify TIA patients at high risk and give effective treatment to prevent stroke occurrence.

The use of brain magnetic resonance imaging (MRI) has shown that some patients with a clinical definition of TIA have neuroimaging evidence of brain injury on diffusion-weighted imaging (DWI). Moreover, recent studies demonstrated that adding imaging information, e.g. ABCD^3^-I score, showed improved risk predictive ability in TIA patients^3–5^. However, without more detailed imaging parameters, such as DWI pattern, the ABCD^3^-I score would have no good predictive ability of stroke in TIA patients with positive DWI.

DWI patterns may suggest differences in pathogenesis, which are predictive for stroke recurrence^6^. For example, multiple acute infarctions are inclined to be caused by a plaque rupture with multiple distal emboli or a cardiac source of embolism^7^. The TIAregistry.org project showed that acute infarctions, especially for multiple infarctions, portended an increased risk of stroke in TIA and minor stroke patients^8^. Recently, the comprehensive stroke recurrence (CSR) model, a neuroimaging-based model including DWI patterns, has been proved that had a high predictive value of recurrent stroke for stroke patients^9^. As a promising risk prediction tool for TIA, CSR model still needs to be validated in different patient population before it can be widely utilized.

Therefore, the aims of our study are: (1) validating the predictive value of the CSR model in TIA patients; (2) comparing the predictive ability of CSR model with ABCD^3^-I score.

## Method

### Study population

The data of our study was from the hospital-based TIA database of the First Affiliated Hospital of Zhengzhou University. The background and methods of the database were described in other articles^3,10^. This prospective cohort study consecutively recruited 1652 TIA patients whose onset time is less than 7 days from October 2010 to August 2017. Patients were diagnosed based on the traditional TIA criteria, an acute loss of focal cerebral or ocular dysfunction lasting less than 24 h attributed to embolic or thrombotic vascular diseases^11^. Patients were excluded if they met the following criteria: (1) unavailable CSR score or ABCD^3^-I score. (2) Failure to complete imaging assessment before stroke occurrence. (3) Lost to follow-up. The research was approved by the ethics committee of the First Affiliated Hospital of Zhengzhou University. The informed consent forms were obtained from all of the patients or their legal proxies.

### Data collection

Trained physicians recorded all detailed baseline data of enrolled TIA patients, including demographics such as age, sex, vascular risk factors such as current smoking, hypertension, diabetes mellitus, dyslipidemia, previous stroke, atrial fibrillation or coronary heart disease, imaging features, and treatment for secondary prevention (antiplatelet agents, anticoagulant, lipid-lowing agents, antihypertension agents and hypoglycemic agents).

All patients without contradictions of magnetic resonance imaging (MRI) were recommended to undergo an etiologic work-up according to a standard protocol at baseline within 7 days of the index TIA. The index TIA was defined as the most recently preceding assessment by a stroke specialist. The standardized work-up included brain MRI (3.0 T, diffusion-weighted imaging [DWI], apparent diffusion coefficient [ADC], T1/T2-weighted imaging, fluid-attenuated inversion recovery [FLAIR] sequences) and arterial examination (at least one assessment of intracranial arteries, and at least one assessment of extracranial arteries). Intracranial artery imaging included 3-dimensional time-of-flight magnetic resonance angiography, computed tomography angiography, or digital subtraction angiography. Extracranial artery imaging included carotid doppler, computed tomography angiography, contrast-enhanced magnetic resonance angiography, or digital subtraction angiography. Two trained readers independently reviewed the imaging data and were blinded to the patients’ baseline and outcome information. Any disagreement was decided by a third reader.

### ABCD^3^-I score and CSR model

The ABCD^3^-I score^12^ is a 13-point scale consisting of six clinical variables (age, blood pressure, clinical features, duration of symptoms, and history of diabetes mellitus, dual TIA [an earlier TIA within 7 days of the index event]) and two imaging findings (ipsilateral ≥ 50% stenosis of internal carotid artery and acute DWI hyperintensity). The CSR model is a scale that totally based on neuroimaging parameters^9^. It is an 8-point scale including five neuroimaging parameters (multiple stage lesion, isolated cortical lesion, WMH, OLI and relevant arterial stenosis; Table 1).

**Table 1:** Comprehensive stroke recurrence (CSR) model.

	CSR model
Multiple stage lesion	3
Isolated cortical lesion	1
Severe white matter hyperintensity	1
**Number of old lacunar infarction**	
0	0
1–2	1
> 2	2
Relevant arterial stenosis	1

Multiple stage lesion (i.e. lesions of varying age) was defined as multiple DWI lesions that met at least 2 of the following: (1) hyperacute: hypointense on ADC and isointense on FLAIR imaging; (2) early acute: hypointense on ADC and hyperintense on FLAIR; (3) late acute to subacute: isointense on ADC and hyperintense on FLAIR. Isolated cortical lesion was defined as DWI lesions that involved only cortical areas without subcortical involvement. WMH was rated on FLAIR imaging using the Fazekas scale in peri-ventricular and subcortical areas, respectively, then summed up the score in two areas and dichotomized into mild (0–2) and severe (3–6) WMH. OLI was initially rated as numbers according to well-defined lesions on MRI (maximum diameter in the axial plane ≥ 3 mm), and then divided them into three categories (OLI, 0; OLI, 1–2 and OLI ≥ 3). Relevant arterial stenosis was defined as ≥ 50% narrowing in the lumen of the intracranial or extracranial artery responsible for the neurological symptoms. The rate of arterial stenosis is calculated based on the North American symptomatic carotid endarterectomy study (NASCET)^13^:

$${\text{The rate of arterial stenosis }} = \, \left( {{\text{D}} - {\text{d}}} \right)/ {\text{D}} \times {1}00\% .$$

D referred to diameter of normal lumen, and d referred to diameter of the narrowest lumen.

### Follow-up and outcome

All registered patients were followed up over the telephone by a neurologist blinded to the clinical information and prognostic scores. All patients who were suspected of having a stroke were followed up via face-to-face interview. The predictive outcome was stroke occurrence within 90 days. Stroke was defined as a rapidly developed clinical symptom of focal (or occasionally global) disturbance of cerebral function, lasting more than 24 h or until death, with no apparent non-vascular cause^14^.

### Statistics analysis

Participants were assigned to the group of positive DWI or negative DWI according to the presence or absence of DWI-identified lesions and analyses were performed in all TIA patients and subgroup of DWI positive or DWI negative patients. We used chi-square test for categorical variables; t-test for normally distributed continuous variables and Mann–Whitney U test for non-normally distributed continuous variables. Cochran-Armitage trend test was performed for patients for each CSR model score. Cumulative event curves were constructed for the end point by the Kaplan–Meier method, and the risk of stroke (stratified according to the score) was compared by the log-rank test. Univariate cox proportional hazards regression modeling was conducted to determine the risk factors associated with stroke occurrence. If there were statistical differences by univariate analysis at baseline, confounding factors were selected to examine the relationship between the risk predictive scores and stroke occurrence using multivariable Cox proportional hazards regression. Associations were presented as hazard ratio (HR) with corresponding 95% confidence interval (CI). To investigate the extent to which CSR model improved the risk assessment of incident stroke in comparison with the ABCD^3^-I score, the C statistics, integrated discrimination improvement (IDI), and net reclassification index (NRI) was calculated.

All statistical analyses were performed using SPSS software for Windows (version 25.0; SPSS). A two-tailed value of *P* < 0.05 was considered statistically significant.

## Results

Of all 1652 consecutive patients with a diagnosis of TIA, 457 patients with unavailable scores (286 with unavailable CSR score, 60 with unavailable ABCD^3^-I score, and 111 with unavailable CSR score and ABCD^3^-I score) and 9 patients lost to 90-day follow-up were excluded. Finally, there were 1186 eligible patients in this study. Comparison of baseline characteristics of the included and excluded groups showed no significant differences (Table S1). Among the 1186 patients, the mean age was 57.28 ± 12.17 years, 719 (60.6%) of patients were men, and 474 (40.0%) patients had positive DWI on MRI. Table 2 showed baseline characteristics in TIA patients. Compared to TIA patients with negative DWI, those with positive DWI were more likely to be men with smoking, have higher ABCD^3^-I and CSR scores, have a history of hypertension and anticoagulant agents takers.

**Table 2:** Baseline characteristics of enrolled TIA participants.

	All TIA patients (n = 1186)	TIA patients with negative DWI (n = 712)	TIA patients with positive DWI (n = 474)	*P* value
Age ≥ 60	531 (44.8%)	312 (43.8%)	219 (46.2%)	0.419
Male	719 (60.6%)	409 (57.4%)	310 (65.4%)	0.006
Current smoking	335 (28.3%)	170 (23.9%)	165 (35.0%)	< 0.001
DWI in 3 days	953 (80.4%)	562 (78.9%)	391 (82.5%)	0.131
**Medical history**				
Hypertension	650 (54.8%)	370 (52.0%)	280 (59.1%)	0.016
Diabetes	207 (17.5%)	113 (15.9%)	94 (19.8%)	0.078
Dyslipidemia	227 (19.1%)	129 (18.1%)	98 (20.7%)	0.273
Coronary heart disease	154 (13.0%)	95 (13.3%)	59 (12.4%)	0.653
Atrial fibrillation	26 (2.2%)	11 (1.5%)	15 (3.2%)	0.062
History of stroke	248 (20.9%)	136 (19.1%)	112 (23.6%)	0.060
ABCD^3^-I score (median, IQR)	5 (4–7)	5 (4–7)	7 (6–9)	< 0.001
CSR score (median, IQR)	1 (0–2)	1 (0–2)	4 (1–6)	< 0.001
**Discharge treatment**				
Antiplatelet agents	1116 (94.1%)	664 (93.3%)	452 (95.4%)	0.133
Anticoagulant	38 (3.2%)	13 (1.8%)	25 (5.3%)	0.001
Lipid-lowering agents	1108 (93.4%)	660 (92.7%)	448 (94.5%)	0.216
Antihypertension agents	398 (33.6%)	239 (33.6%)	159 (33.5%)	0.993
Hypoglycemic agents	230 (19.4%)	126 (17.7%)	104 (21.9%)	0.070

*TIA* transient ischemic attack, *DWI* diffusion-weighted imaging, *CSR* comprehensive stroke recurrence.

**Figure 1:**
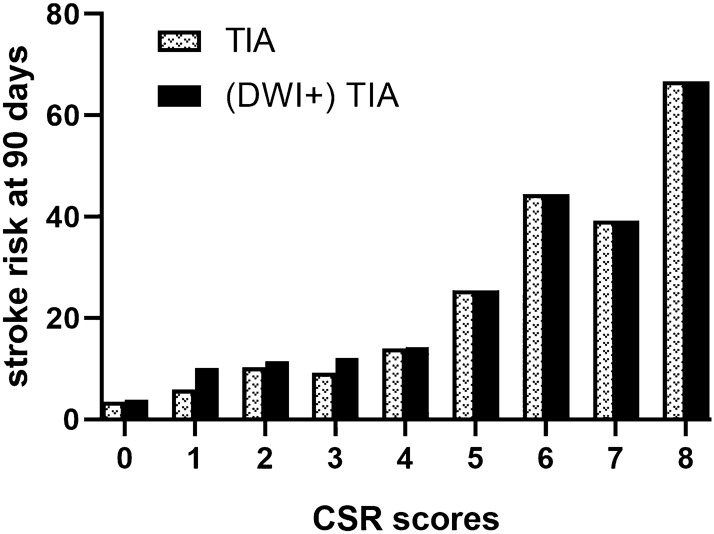
Stroke risk at 90 days according to CSR score.

**Figure 2:**
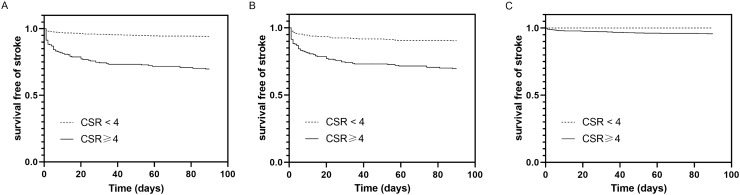
(**A**) Kaplan–Meier curves of TIA patients stratified according to the CSR model at 90 days (log-rank test = 121.70, *P* < 0.001); (**B**) Kaplan–Meier curves of DWI-positive subjects stratified according to the CSR model at 90 days (log-rank test = 33.75, *P* < 0.001); (**C**) Kaplan–Meier curves of DWI-negative subjects stratified according to the CSR model at 90 days (log-rank test = 6.89, *P* = 0.142).

We analyzed the incidence of stroke at different follow-up periods. In TIA patients, the stroke rate at day 2, 7 and 90 was 3.5%, 5.2% and 9.9%, respectively. In those with positive DWI, the stroke rate at day 2, 7, and 90 was 7.0%, 10.1% and 18.4%. In those with negative DWI, the stroke rate at day 2, 7 and 90 was 1.1%, 2.0% and 4.4%. Figure 1 showed the stroke risk according to different CSR score in TIA patients and TIA patients with positive DWI, which illustrated an overall increase in the rate of stroke with increasing CSR score. A linear trend for occurrence rates was observed with the Cochran-Armitage trend test in TIA patients (Z =  − 11.627; *P* < 0.0001) and TIA patients with positive DWI (Z =  − 6.9986; *P* < 0.0001). TIA patients were categorized into low CSR score group (0–3) and high CSR score group (4–8) based on optimal cut-off point which was calculated by the maximum Youden index. Survival-free-of stroke curves categorized by CSR model were shown in Fig. 2. In TIA patients, survival curve was significantly different between the low-risk and high-risk categories (log-rank test = 121.70; *P* < 0.001). Similar result was found in TIA patients with positive DWI (log-rank test = 33.75; *P* < 0.001). But the trend was not found in those with negative DWI.

Table 3 showed the clinical characteristics and risk factors associated with stroke in TIA patients. Those who undergone stroke were more likely to have history of hypertension, higher ABCD^3^-I score and CSR score in TIA patients. In those with positive DWI, those who undergone stroke were more likely to be women, have history of hypertension and stroke, higher ABCD^3^-I score and CSR score. Final multivariate analysis revealed that history of hypertension (HR 1.547; 95% CI 1.031–2.32), high ABCD^3^-I score (HR 1.216; 95% CI 1.116–1.326) and CSR score ≥ 4 (HR 3.708; 95% CI 2.487–5.53) were independent risk factors of stroke occurrence in TIA patients. In TIA patients with positive DWI, the final multivariate analysis showed that history of hypertension (HR 1.795; 95% CI 1.094–2.945), history of stroke (HR 1.641; 95% CI 1.040–2.590), high ABCD^3^-I score (HR 1.181; 95% CI 1.058–1.319), and CSR score ≥ 4 (HR 3.075; 95% CI 1.942–4.868) were independent risk factors of stroke occurrence.

**Table 3:** Baseline characteristics and risk factors of patients with and without recurrence at 90 days.

	Stroke occurrence at 90 days								
	TIA patients (n = 1186)			TIA patients with positive DWI (n = 474)			TIA patients with negative DWI (n = 712)		
	No (n = 1068)	Yes (n = 118)	*P* value	No (n = 387)	Yes (n = 87)	*P* value	No (n = 681)	Yes (n = 31)	*P* value
Age ≥ 60 years	469 (43.9%)	62 (52.5%)	0.074	172 (44.4%)	47 (54.0%)	0.105	297 (43.6%)	15 (48.4%)	0.600
Male	646 (60.5%)	73 (61.9%)	0.771	260 (67.2%)	50 (57.5%)	0.085	386 (56.7%)	23 (74.2%)	0.054
Current smoking	295 (27.7%)	40 (33.9%)	0.158	137 (35.7%)	28 (32.2%)	0.537	158 (23.2%)	12 (38.7%)	0.078
DWI in 3 days	857 (80.2%)	96 (81.4%)	0.773	319 (82.4%)	72 (82.8%)	0.942	538 (79%)	24 (77.4%)	0.833
**Medical history**									
Hypertension	566 (53.0%)	84 (71.2%)	< 0.001	215 (55.6%)	65 (74.7%)	0.001	351 (51.5%)	19 (61.3%)	0.288
Diabetes	186 (17.4%)	21 (17.8%)	0.918	77 (19.9%)	17 (19.5%)	0.940	109 (16.0%)	4 (12.9%)	0.833
Dyslipidemia	203 (19.0%)	24 (20.3%)	0.727	83 (21.4%)	15 (17.2%)	0.381	120 (17.6%)	9 (29.0%)	0.107
Coronary heart disease	138 (12.9%)	16 (13.6%)	0.845	48 (12.4%)	11 (12.6%)	0.951	90 (13.2%)	5 (16.1%)	0.641
Atrial fibrillation	25 (2.3%)	1 (0.8%)	0.472	14 (3.6%)	1 (1.1%)	0.396	11 (1.6%)	0 (0%)	–
History of stroke	217 (20.3%)	31 (26.3%)	0.131	85 (22.0%)	27 (31.0%)	0.072	132 (19.4%)	4 (12.9%)	0.507
**Clinical symptom**									
Unilateral weakness	122 (11.4%)	16 (13.6%)	< 0.001	50 (12.9%)	11 (12.6%)	0.067	72 (10.6%)	5 (16.1%)	0.208
Speech disturbance without weakness	569 (53.3%)	82 (69.5%)		240 (62.0%)	64 (73.6%)		329 (48.3%)	18 (58.1%)	
**Duration**									
< 10 min	441 (41.3%)	32 (27.1%)	0.004	144 (37.2%)	24 (27.6%)	0.125	297 (43.6%)	8 (25.8%)	0.124
10–59 min	411 (38.5%)	63 (53.4%)		164 (42.4%)	47 (54.0%)		247 (36.3%)	16 (51.6%)	
≥ 60 min	216 (20.2%)	23 (19.5%)		79 (20.4%)	16 (18.4%)		137 (20.1%)	7 (22.6%)	
ABCD^3^-I score (median, IQR)	5 (4–7)	7 (6–9)	< 0.001	7 (6–9)	8 (7–10)	< 0.001	4 (3–6)	5 (4–6)	0.092
Multiple stage lesion	119 (11.1%)	56 (47.5%)	< 0.001	119 (30.7%)	56 (64.4%)	< 0.001	…	…	–
Isolated cortical lesion	161 (15.1%)	46 (39.0%)	< 0.001	161 (41.6%)	46 (52.9%)	0.055	…	…	–
Severe WMH	193 (18.1%)	41 (34.7%)	< 0.001	104 (26.9%)	37 (42.5%)	0.004	89 (13.1%)	4 (12.9%)	0.979
**Number of OLI**									
0	555 (52.0%)	32 (27.1%)	< 0.001	177 (45.7%)	19 (21.8%)	< 0.001	378 (55.5%)	13 (41.9%)	0.066
1–2	369 (34.6%)	54 (45.8%)		148 (38.2%)	44 (50.6%)		221 (32.5%)	10 (32.3%)	
> 2	144 (13.5%)	32 (27.1%)		62 (16.0%)	24 (27.6%)		82 (12.0%)	8 (25.8%)	
Relevant arterial stenosis	185 (17.3%)	57 (48.3%)	< 0.001	184 (47.5%)	57 (65.5%)	0.002	1 (0.1%)	0 (0%)	–
CSR score (median, IQR)	1 (0–2)	4 (1–6)	< 0.001	2 (1–4)	5 (3–6)	< 0.001	0 (0–1)	1 (1–2)	0.078
**Discharge treatment**									
Antiplatelet agents	1006 (96.3%)	110 (95.7%)	0.743	371 (95.9%)	81 (93.1%)	0.268	635 (93.2%)	29 (93.5%)	0.948
Anticoagulant	35 (3.3%)	3 (2.5%)	0.877	22 (5.7%)	3 (3.4%)	0.563	13 (1.9%)	0 (0%)	–
Lipid-lowering agents	996 (93.3%)	112 (94.9%)	0.491	364 (94.1%)	84 (96.6%)	0.507	632 (92.8%)	28 (90.3%)	0.868
Antihypertension agents	356 (33.3%)	42 (35.6%)	0.622	127 (32.8%)	32 (36.8%)	0.479	229 (33.6%)	10 (32.3%)	0.875
Hypoglycemic agents	206 (19.3%)	24 (20.3%)	0.784	85 (22.0%)	19 (21.8%)	0.980	121 (17.8%)	5 (16.1%)	0.815

Statistical analysis not possible owing to absence of events in the group of TIA patients with negative DWI.

*TIA* transient ischemic attack, *DWI* diffusion-weighted imaging, *CSR* comprehensive stroke recurrence, *OLI* old lacunar infarction.

**Figure 3:**
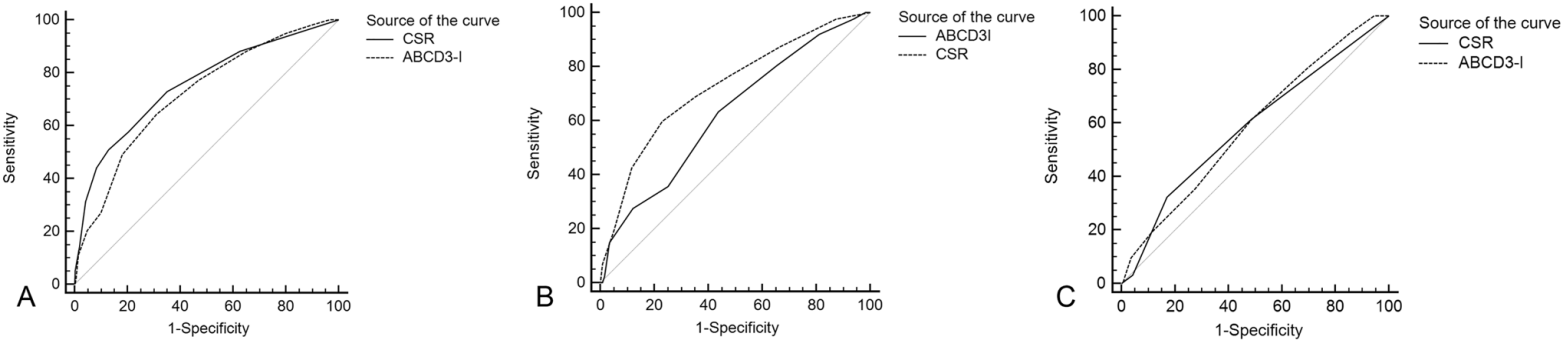
Ninety-day receiver-operating characteristic curves as a predictive value of the CSR score and ABCD^3^-I score. (**A**) The C statistic of CSR model (0.754; 95% CI 0.729–0.778) was similar with that of ABCD^3^-I score (0.717; 95% CI 0.691–0.743, Z = 1.400; P = 0.1616) in TIA patients; (**B**) CSR score showed better discrimination (Z = 2.294, P = 0.0245) with the C statistic of 0.725 (95% CI 0.683–0.765) than ABCD^3^-I score (0.626; 95% CI 0.581–0.670) in DWI-positive subjects. (**C**) The C statistic of CSR model (0.585; 0.548–0.622) was similar with that of ABCD^3^-I score (0.588; 0.551–0.624, Z = 0.0362; P = 0.9711) in TIA patients with negative DWI.

Comparing the predictive ability of ABCD^3^-I score and CSR model in TIA patients using the receiver-operating characteristic curve analysis (Fig. 3). The area under the curve (AUC) was 0.717 (95% CI 0.691–0.743) by the ABCD^3^-I score and 0.754 (95% CI 0.729–0.778) by the CSR score for the prediction of 90-day stroke occurrence. The comparison of the AUC was performed by Z test, which revealed that the C statistic for CSR model was higher than that for the ABCD^3^-I score (Z = 1.400; *P* = 0.1616) but the result was not statistically significant. Similar results were found in those with negative DWI. However, the comparison of the two scores was statistically significant in TIA patients with positive DWI. The analysis suggested that the AUC was 0.626 (95% CI 0.581–0.670) by the ABCD^3^-I score and 0.725 (95% CI 0.683–0.765) by the CSR score for the prediction of 90-day stroke occurrence. The Z test revealed that the C statistic for CSR model was higher than that for the ABCD^3^-I score (Z = 2.294; *P* = 0.0245). The discriminatory power and risk reclassification also appeared to be substantially better (IDI, 7.9%, P < 0.001; category NRI, 55.1%, P < 0.001).

## Discussion

The major findings of this study were the following: (1) usefulness of CSR model to predict the 90-day risk of stroke after TIA was validated; (2) the CSR model were superior to the ABCD^3^-I score in predicting the risk of 90-day stroke in TIA patients with positive DWI. In this study, the 90-day stroke rate was 9.9% in TIA patients, which was identical to the pooled risk (9.2%) based on a random effects model in a systematic review and meta-analysis^15^. However, the TIAregistry.org project reported that the risk of stroke occurrence after TIA was 3.7% at 90 days^8^ and another meta-analysis of recent intervention studies reported that the pooled stroke risk was 3.42% at 90 days^16^. One of possible reasons for the discrepancy may be that only hospitalized patients were enrolled in our study, which had high incidence of stroke compared with patients from clinic or emergency department. Moreover, the definition of TIA was distinguishing in different studies. In our study, the definition of TIA was time-based, however, the TIAregistry.org project^8^ defined TIA as new symptomatic neurologic deterioration lasting less than 24 h with no new infarction on neuroimaging, which was tissue-based definition. This definition did not take TIA patients with positive DWI into account, which was another potential reason of low rate of stroke occurrence. Correspondingly, the stroke risk for TIA with negative DWI was 4.4% in our study, which are consistent with these studies.

Recent studies demonstrated that the combination of neuroimaging and vascular information could further improve the predictive value of stroke after TIA^17–19^. ABCD^3^-I score provided risk estimates based on neuroimaging in addition to clinical predictors, which was widely used to stratify the risk factors of stroke in many studies^20,21^. In our study, the ABCD^3^-I score showed a good predictive value of stroke in TIA patients, which was similar to previous studies. And CSR model, based on more detailed neuroimaging, provided good discrimination for risk prediction of early stroke among TIA patients in our study.

An abnormal DWI has been validated as increasing the early stroke risk in many studies, especially DWI patterns^12,18^, such as infarction numbers^8,22^, different infarction stages^23,24^, and infarction location^17,18^. For example, Amarenco et al.^8^ showed that acute multiple infarctions portended an increased risk of stroke in TIA and minor stroke patients. Sylaja et al.^25^ reported that patients with multiple DWI lesions of different age were at 3.6-times-higher risk of having new lesions at 30 days than those having lesions of the same age. Purroy et al.^26^ demonstrated that single cortical lesions were also associated with cardioembolism, whereas subcortical acute lesions were associated with recurrent stroke. CSR model, a neuroimaging-based model including DWI patterns, showed better predictive value than ABCD^3^-I score for TIA patients with positive DWI In our present study. The superiority in predicting the subsequent stroke of CSR model could be explained by detailed imaging features of infarction provided the prognostic information that related to the underlying stroke mechanism. For instance, multiple infarcts of different ages indicated repeated ischemic events at different time points and had prognostic significance because they signify one or more “unstable” underlying causative lesions^19^. Cortical lesion was usually related to cardioembolism and cryptogenic with pathogenesis of embolism^22,23^. OLI or severe WMH indicated small vessel disease which could cause hostile brain conditions that were more vulnerable to ischemic insults^24,27^. In a study of 959 patients with first-ever acute ischemic stroke (AIS), the CSR model applied within 7 days of AIS provided an AUC of 0.81 for 2-year risk of recurrent stroke, and internal and external validation analysis also showed good predictive power^9^. Our study provided further evidence that the CSR model could predict stroke risk in TIA patients with positive DWI. Therefore, CSR model could stratify TIA patients, especially those with positive DWI. In clinical practice, ABCD^3^-I score could be used for initial assessment for its easier evaluation method, and CSR model could stratify DWI-positive patients further based on detailed neuroimaging findings and determine individualized secondary prevention strategies for the purpose of reducing the risk of stroke after TIA.

The study has several limitations. First, a total of 466 excluded patients (28.2%) would cause selection bias. As post-hoc analysis of prospective research, this limitation cannot be avoided. Incomplete MRI imaging sequences or imaging examinations in other hospitals lead to unavailable CSR score. However, baseline characteristics of included and excluded patients in the study were similar. Second, we evaluated only hospitalized patients at a single center, which could lead to selective bias. These findings need further validation by multicenter prospective studies.

## Conclusion

CSR model and ABCD^3^-I score had good predictive value for predicting the risk of 90-day stroke in patients with definite TIA. Moreover, CSR model was superior to ABCD^3^-I score in predicting the risk of 90-day stroke in TIA patients with positive DWI. Using CSR model, we could stratify high-risk TIA patients further.

### Ethical statement

The study was performed according to the guidelines of the Helsinki Declaration. The study was undertaken with the understanding and written consent of each subject and was approved by the Ethics Committee of the First Affiliated Hospital of Zhengzhou University.

## Supplementary Information

Supplementary Table S1.

## Data Availability

Data are available to researchers on request for purposes of reproducing the results or replicating the procedure by directly contacting the corresponding author.

## References

[1] von Weitzel-Mudersbach P, Andersen G, Hundborg HH, Johnsen SP (2013). Transient ischemic attack and minor stroke are the most common manifestations of acute cerebrovascular disease: A prospective, population-based study—The Aarhus TIA study. Neuroepidemiology.

[2] Giles MF, Rothwell PM (2007). Risk of stroke early after transient ischaemic attack: A systematic review and meta-analysis. Lancet Neurol..

[3] Song B (2013). Validation of the ABCD3-I score to predict stroke risk after transient ischemic attack. Stroke.

[4] Zhao M (2017). Comparison of stroke prediction accuracy of ABCD2 and ABCD3-I in patients with transient ischemic attack: A meta-analysis. J. Stroke Cerebrovasc. Dis..

[5] Knoflach M (2016). Predictive value of ABCD2 and ABCD3-I scores in TIA and minor stroke in the stroke unit setting. Neurology.

[6] Engelter ST (2012). Optimizing the risk estimation after a transient ischaemic attack—The ABCDE⊕ score. Eur. J. Neurol..

[7] Hyafil F (2014). Rupture of nonstenotic carotid plaque as a cause of ischemic stroke evidenced by multimodality imaging. Circulation.

[8] Amarenco P (2016). One-year risk of stroke after transient ischemic attack or minor stroke. N. Engl. J. Med..

[9] Nam KW, Kwon HM, Lim JS, Han MK, Lee YS (2017). Clinical relevance of abnormal neuroimaging findings and long-term risk of stroke recurrence. Eur. J. Neurol..

[10] Song B (2019). Efficacy of high-intensity statin use for transient ischemic attack patients with positive diffusion-weighted imaging. Sci. Rep..

[11] Hatano S (1976). Experience from a multicentre stroke register: A preliminary report. Bull. World Health Organ..

[12] Merwick A (2010). Addition of brain and carotid imaging to the ABCD^2^ score to identify patients at early risk of stroke after transient ischaemic attack: A multicentre observational study. Lancet Neurol..

[13] Barnett HJM (1991). Beneficial effect of carotid endarterectomy in symptomatic patients with high-grade carotid stenosis. N. Engl. J. Med..

[14] Whisnant JP (1990). Classification of cerebrovascular diseases III. Stroke.

[15] Wu CM (2007). Early risk of stroke after transient ischemic attack: A systematic review and meta-analysis. Arch. Intern. Med..

[16] Valls J (2017). A current estimation of the early risk of stroke after transient ischemic attack: A systematic review and meta-analysis of recent intervention studies. Cerebrovasc. Dis..

[17] Jung JM (2012). Predictors of recurrent stroke in patients with symptomatic intracranial arterial stenosis. Stroke.

[18] Wang G (2019). Does all single infarction have lower risk of stroke recurrence than multiple infarctions in minor stroke?. BMC Neurol..

[19] Wardlaw JM (2014). Vascular risk factors, large-artery atheroma, and brain white matter hyperintensities. Neurology.

[20] Purroy F (2013). Predictive value of brain and vascular imaging including intracranial vessels in transient ischaemic attack patients: External validation of the ABCD3-I score. Eur. J. Neurol..

[21] Dai Q (2015). From clinical to tissue-based dual TIA: Validation and refinement of ABCD3-I score. Neurology.

[22] Lee DK, Kim JS, Kwon SU, Yoo SH, Kang DW (2005). Lesion patterns and stroke mechanism in atherosclerotic middle cerebral artery disease: Early diffusion-weighted imaging study. Stroke.

[23] Bang OY (2005). Specific DWI lesion patterns predict prognosis after acute ischaemic stroke within the MCA territory. J. Neurol. Neurosurg. Psychiatry.

[24] Lau KK (2017). Total small vessel disease score and risk of recurrent stroke: Validation in 2 large cohorts. Neurology.

[25] Sylaja PN (2007). Acute ischemic lesions of varying ages predict risk of ischemic events in stroke/TIA patients. Neurology.

[26] Purroy F (2011). Patterns of diffusion-weighted magnetic resonance imaging associated with etiology improve the accuracy of prognosis after transient ischaemic attack. Eur. J. Neurol..

[27] Song TJ (2017). Total cerebral small-vessel disease score is associated with mortality during follow-up after acute ischemic stroke. J. Clin. Neurol..

