# Supplementary material for: Validation of CSR model to predict stroke risk after transient ischemic attack
Source: Sci Rep. 2022 Jan 12;12:604. doi: 10.1038/s41598-021-04405-2 (PMC8755815; doi:10.1038/s41598-021-04405-2)
Supplement: Supplementary file 1 — Supplementary Table S1. [file 41598_2021_4405_MOESM1_ESM.docx]

Table S1 Baseline characteristics of excluded and included participants.

|  | Excluded (n=466) | Included (n=1186) | *P* value |
| --- | --- | --- | --- |
| Age≥60 | 205(44.0%) | 531(44.8%) | 0.774 |
| Male | 285(61.2%) | 719(60.6%) | 0.841 |
| Current smoking | 114(24.6%) | 335(28.3%) | 0.122 |
| Medical history | | | |
| Hypertension | 254(54.5%) | 650(54.8%) | 0.912 |
| Diabetes | 77(16.5%) | 207(17.5%) | 0.652 |
| Dyslipidemia | 100(21.5%) | 227(19.1%) | 0.287 |
| Coronary heart disease | 62(13.3%) | 154(13.0%) | 0.862 |
| Atrial fibrillation | 8(1.7%) | 26(2.2%) | 0.540 |
| History of stroke | 96(20.6%) | 248(20.9%) | 0.889 |
| ABCD^3^-I score (median, IQR) | 5(4-7) | 5(4-7) | 0.028 |
| CSR score (median, IQR) | 1(0-2) | 1(0-2) | 0.386 |
| Discharge treatment | | | |
| Antiplatelet Therapy | 434(93.1%) | 1116(94.1%) | 0.463 |
| Anticoagulant | 15(3.2%) | 38(3.2%) | 0.988 |
| Lipid-lowering agents | 426(91.4%) | 1108(93.4%) | 0.154 |
| Antihypertension agents | 168(36.1%) | 398(33.6%) | 0.337 |
| Hypoglycemic agents | 92(19.7%) | 230(19.4%) | 0.872 |

Abbreviations: CSR=comprehensive stroke recurrence.
